# Supplementary material for: Assembly and substrate recognition of curli biogenesis system
Source: Nat Commun. 2020 Jan 13;11:241. doi: 10.1038/s41467-019-14145-7 (PMC6957492; doi:10.1038/s41467-019-14145-7)
Supplement: Supplementary file 1 — Supplemetray information [file 41467_2019_14145_MOESM1_ESM.pdf]

Supplementary Information

Assembly and Substrate Recognition of Curli Biogenesis System

Yan et al.

Supplementary Table 1. Primer sequences used for experiments.

Constructs	Primers	Sequences
**CsgEFG_Cstrep**	Forward_end	GTGCTGCATATGAAACGTTATTTACGCTGGATTGTG
	Reverse_end	CCTTCAGGATCCTTATTTTTCGAACTGCGGGTGGCTCCAAGCGCTGGATTCCGGTGGAACCGACATGTGGCGGTATTTC
**CsgG_Cstrep**	Forward_end	GTGCTGCATATGCAGCGCTTATTTCTTTTGG
	Reverse_end	CCTTCAGGATCCTTATTTTTCGAACTGCGGGTGGCTCCAAGCGCTGGATTCCGGTGGAACCGACAT
**MBP_Chis**	Forward_end	GTGCTGCATATGAAAATCGAAGAAGGTAAACTGGTAATC
	Reverse_end	CCTTCAGGATCCTTAGTGATGATGATGATGATGAGCGCTAGTCTGCGCGTCTTTCAG
**CsgF-MBP_Chis**	Forward_end	GTGCTGCATATGGGAACCATGACTTTCCAGTTC
	Reverse_end	CCTTCAGGATCCTTAGTGATGATGATGATGATGAGCGCTAGTCTGCGCGTCTTTCAG
	Forward_intermediate	CAAAATAACTCAACCGATTTTAAAATCGAAGAAGGTAAAC
	Reverse_intermediate	GTTTACCTTCTTCGATTTTAAAATCGGTTGAGTTATTTTG
**CsgF$_N$-MBP_Chis**	Forward_end	GTGCTGCATATGGGAACCATGACTTTCCAGTTC
	Reverse_end	CCTTCAGGATCCTTAGTGATGATGATGATGATGAGCGCTAGTCTGCGCGTCTTTCAG
	Forward_intermediate	CTCTTATAAAGATCCGAGCAAAATCGAAGAAGGTAAACTG
	Reverse_intermediate	CAGTTTACCTTCTTCGATTTTGCTCGGATCTTTATAAGAG

Constructs	Primers	Sequences
**$CsgF_C$-MBP_Chis**	Forward_end	GTGCTGCATATGTATAACGATGACTTTGGTATTGAAAC
	Reverse_end	CCTTCAGGATCCTTAGTGATGATGATGATGATGAGCGCTAGTCTGCGCGTCTTTCAG
	Forward_intermediate	CAAAATAACTCAACCGATTTTAAAATCGAAGAAGGTAAAC
	Reverse_intermediate	GTTTACCTTCTTCGATTTTAAAATCGGTTGAGTTATTTTG
**MBP_Nstrep**	Forward_end	GTGCTGCATATGTGGAGCCACCCGCAGTTCGAAAAAAGCGCTAAAATCGAAGAAGG
	Reverse_end	CCTTCAGGATCCTTAAGTCTGCGCGTCTTTCAGGGCTTC
**MBP-CsgF_Nstrep**	Forward_end	GTGCTGCATATGTGGAGCCACCCGCAGTTCGAAAAAAGCGCTAAAATCGAAGAAGG
	Reverse_end	CCTTCAGGATCCTTAAAAATCGGTTGAGTTATTTTG
	Forward_intermediate	CTGAAAGACGCGCAGACTAGCGCTGGAACCATGACTTTCC
	Reverse_intermediate	GGAAAGTCATGGTTCCAGCGCTAGTCTGCGCGTCTTTCAG
**MBP-$CsgF_N$_Nstrep**	Forward_end	GTGCTGCATATGTGGAGCCACCCGCAGTTCGAAAAAAGCGCTAAAATCGAAGAAGG
	Reverse_end	CCTTCAGGATCCTTAGCTCGGATCTTTATAAGAG
	Forward_intermediate	CTGAAAGACGCGCAGACTAGCGCTGGAACCATGACTTTCC
	Reverse_intermediate	GGAAAGTCATGGTTCCAGCGCTAGTCTGCGCGTCTTTCAG

Constructs	Primers	Sequences
**MBP-CsgF$_C$_Nstrep**	Forward_end	GTGCTGCATATGTGGAGCCACCCGCAGTTCGAAAAAAGCGCTAAAATCGAAGAAGG
	Reverse_end	CCTTCAGGATCCTTAAAAATCGGTTGAGTTATTTTG
	Forward_intermediate	CTGAAAGACGCGCAGACTAGCGCTTATAACGATGACTTTGG
	Reverse_intermediate	CCAAAGTCATCGTTATAAGCGCTAGTCTGCGCGTCTTTCAG
**CsgB-MBP_Chisflag**	Forward_end	GTGCTGCATATGGCAGGTTATGATTTAGCT
	Reverse_end-1	CTTGTAATCAGCGCTGTGATGATGATGATGATGAGCGCTAGTCTGCGCGTCTTTCAG
	Reverse_end-2	CCTTCAGGATCCTTACTTATCGTCGTCATCCTTGTAATCAGCGCTGTGATGATG
	Forward_intermediate	CTATTCGCGTGACACAACGTAGCGCTAAAATCGAAGAAGGTAAAC
	Reverse_intermediate	GTTTACCTTCTTCGATTTTAGCGCTACGTTGTGTCACGCGAATAG
**MBP-CsgB_Nstrep**	Forward_end	GTGCTGCATATGTGGAGCCACCCGCAGTTCGAAAAAAGCGCTAAAATCGAAGAAGG
	Reverse_end	CCTTCAGGATCCTTATTAACGTTGTGTCACGCGAATAGC
	Forward_intermediate	CTGAAAGACGCGCAGACTGCAGGTTATGATTTAGCT
	Reverse_intermediate	AGCTAAATCATAACCTGCAGTCTGCGCGTCTTTCAG

Constructs	Primers	Sequences
**CsgA-MBP_Chisflag**	Forward_end	GTGCTGCATATGGGTGTTGTTCCTCAGTACGGC
	Reverse_end	CCTTCAGGATCCTTACTTATCGTCGTCATCCTTGTAATCAGCGCTGTGATGATG
	Forward_intermediate	GCGACCGCTCATCAGTACGGCAAAATCGAAGAAGGTAAAC
	Reverse_intermediate	GTTTACCTTCTTCGATTTTGCCGTACTGATGAGCGGTCGC
**CsgF$_N$-MBP_Chisflag**	Forward_end	GTGCTGCATATGGGAACCATGACTTTCCAGTTC
	Reverse_end	CCTTCAGGATCCTTACTTATCGTCGTCATCCTTGTAATCAGCGCTGTGATGATG
	Forward_intermediate	CTCTTATAAAGATCCGAGCAAAATCGAAGAAGGTAAACTG
	Reverse_intermediate	CAGTTTACCTTCTTCGATTTTGCTCGGATCTTTATAAGAG
**MBP_Chisflag**	Forward_end	GTGCTGCATATGAAAATCGAAGAAGGTAAACTGGTAATC
	Reverse_end	CCTTCAGGATCCTTACTTATCGTCGTCATCCTTGTAATCAGCGCTGTGATGATG
**CsgF$_N$-MBP_Chisflag (R8A)**	Forward_end	GTGCTGCATATGGGAACCATGACTTTCCAGTTCGCGAATCCAAACTTTGGTGGTAACCC
	Reverse_end	CCTTCAGGATCCTTACTTATCGTCGTCATCCTTGTAATCAGCGCTGTGATGATG
**CsgF$_N$-MBP_Chisflag (N9A)**	Forward_end	GTGCTGCATATGGGAACCATGACTTTCCAGTTCCGTGCGCCAAACTTTGGTGGTAACCC
	Reverse_end	CCTTCAGGATCCTTACTTATCGTCGTCATCCTTGTAATCAGCGCTGTGATGATG

Constructs	Primers	Sequences
**$CsgF_N$-MBP_Chisflag (N11A)**	Forward_end	GTGCTGCATATGGGAACCATGACTTTCCAGTTCCGTAATCCAGCGTTTGGTGGTAACCC
	Reverse_end	CCTTCAGGATCCTTACTTATCGTCGTCATCCTTGTAATCAGCGCTGTGATGATG
**$CsgF_N$-MBP_Chisflag (F5D)**	Forward_end	GTGCTGCATATGGGAACCATGACTGATCAGTTCCGTAATCCAAACTTTGGTGGTAACCCAAATAATG
	Reverse_end	CCTTCAGGATCCTTACTTATCGTCGTCATCCTTGTAATCAGCGCTGTGATGATG
**$CsgF_N$-MBP_Chisflag (F7D)**	Forward_end	GTGCTGCATATGGGAACCATGACTTTCCAGGATCGTAATCCAAACTTTGGTGGTAACCCAAATAATG
	Reverse_end	CCTTCAGGATCCTTACTTATCGTCGTCATCCTTGTAATCAGCGCTGTGATGATG
**$CsgF_N$-MBP_Chisflag (F12D)**	Forward_end	GTGCTGCATATGGGAACCATGACTTTCCAGTTCCGTAATCCAAACGATGGTGGTAACCCAAATAATG
	Reverse_end	CCTTCAGGATCCTTACTTATCGTCGTCATCCTTGTAATCAGCGCTGTGATGATG
**$CsgF_N$-MBP_Chisflag (F21D)**	Forward_end-1	GTGCTGCATATGGGAACCATGACTTTCCAGTTCCGTAATCCAAACTTTGGTGGTAAC
	Forward_end-2	CCAAACTTTGGTGGTAACCCAAATAATGGCGCTGATTTATTAAATAGCGCTCAGGCCC
	Reverse_end	CCTTCAGGATCCTTACTTATCGTCGTCATCCTTGTAATCAGCGCTGTGATGATG
**$CsgF_N$-MBP_Chisflag (L22D)**	Forward_end-1	GTGCTGCATATGGGAACCATGACTTTCCAGTTCCGTAATCCAAACTTTGGTGGTAAC
	Forward_end-2	CCAAACTTTGGTGGTAACCCAAATAATGGCGCTTTTGATTTAAATAGCGCTCAGGCCC
	Reverse_end	CCTTCAGGATCCTTACTTATCGTCGTCATCCTTGTAATCAGCGCTGTGATGATG

Constructs	Primers	Sequences
**CsgF$_N$-MBP_Chisflag (L23D)**	Forward_end-1	GTGCTGCATATGGGAACCATGACTTTCCAGTTCCGTAATCCAAACTTTGGTGGTAAC
	Forward_end-2	CCAAACTTTGGTGGTAACCCAAATAATGGCGCTTTTTTAGATAATAGCGCTCAGGCCC
	Reverse_end	CCTTCAGGATCCTTACTTATCGTCGTCATCCTTGTAATCAGCGCTGTGATGATG
**CsgE-Cstrep**	Forward_end	GTGCTGCATATGGCCGTTGAGGTAGAAGTCCCGGGA
	Reverse_end	CCTTCAGGATCCTTATTTTTCGAACTGCGGGTGGCTCCAAGCGCTGAATTCATCATGCGC
**CsgA$_{N22}$-MBP_Chisflag**	Forward_end	GTGCTGCATATGGGTGTTGTTCCTCAGTACGGC
	Reverse_end	CCTTCAGGATCCTTACTTATCGTCGTCATCCTTGTAATCAGCGCTGTGATGATG
	Forward_intermediate	GTAATAATAGCGGCCCAAATGGCAAAATCGAAGAAGGTAAAC
	Reverse_intermediate	GTTTACCTTCTTCGATTTTGCCATTTGGGCCGCTATTATTAC
**CsgA$_{C1-5}$-MBP_Chisflag**	Forward_end	GTGCTGCATATGTCTGAGCTGAACATTTACCAGTAC
	Reverse_end	CCTTCAGGATCCTTACTTATCGTCGTCATCCTTGTAATCAGCGCTGTGATGATG
**CsgE-Chis**	Forward_end	GTGCTGCATATGGCCGTTGAGGTAGAAGTCCCGGGA
	Reverse_end	CCTTCAGGATCCTTAGTGATGATGATGATGATGAGCGCTGAATTCATCATGCGCCAAATCGCC

Constructs	Primers	Sequences
**$CsgA_{N22}$-PhoA-Cstrep**	Forward_end	GTGCTGCATATGAAACTTTTAAAAGTAGCAGCAATTG
	Reverse_end	CCTTCAGGATCCTTATTTTTCGAACTGCGGGTGGCTCCAAGCGCTTTTCAGCCCCAGAGCGGC
	Forward_intermediate	GTAATAATAGCGGCCCAAATGTGACAAAAGCCCGGAC
	Reverse_intermediate	GTCCGGGCTTTTGTCACATTTGGGCCGCTATTATTAC
**CsgEFG_Chis**	Forward_end	GTGCTGCATATGAAACGTTATTTACGCTGGATTGTG
	Reverse_end	CCTTCAGGATCCTTAGTGATGATGATGATGATGAGCGCTGGATTCCGGTGGAACCGACATGTGGCGGTATTTCAC
**CsgEFG_Chis flag**	Forward_end	GTGCTGCATATGAAACGTTATTTACGCTGGATTGTG
	Reverse_end	CCTTCAGGATCCTTACTTATCGTCGTCATCCTTGTAATCAGCGTGATGATGATGATGATGAG
**PhoA_Cstrep**	Forward_end	GTGCTGCATATGAAACAAAGCACTATTGCACTGGCACTC
	Reverse_end	CCTTCAGGATCCTTATTTTTCGAACTGCGGGTGGCTCCAAGCGCTTTTCAGCCCCAGAGCGGC
**$CsgA_{N6}$-PhoA-Cstrep**	Forward_end	GTGCTGCATATGAAACTTTTAAAAGTAGCAGCAATTG
	Reverse_end	CCTTCAGGATCCTTATTTTTCGAACTGCGGGTGGCTCCAAGCGCTTTTCAGCCCCAGAGCGGC
	Forward_intermediate	GCAGGTGTTGTTCCTCAGTACGTGACAAAAGCCCGGACACCA
	Reverse_intermediate	TGGTGTCCGGGCTTTTGTCACGTACTGAGGAACAACACCTGC

Constructs	Primers	Sequences
**CsgF**	Forward_end	GTGCTGCATATGCGTGTCAAACATGCAGTAGTTC
	Reverse_end	CCTTCAGGATCCTTAAAAATCGGTTGAGTTATTTTG
**CsgF**$_N$	Forward_end	GTGCTGCATATGCGTGTCAAACATGCAGTAGTTC
	Reverse_end	CCTTCAGGATCCTTAGCTCGGATCTTTATAAGAGTTTTG
**CsgF**$_C$	Forward_end	GTGCTGCATATGCGTGTCAAACATGCAGTAGTTC
	Reverse_end	CCTTCAGGATCCTTAAAAATCGGTTGAGTTATTTTG
	Forward_intermediate	CCATTAAGTTGGGCTGGATATAACGATGACTTTGGT
	Reverse_intermediate	ACCAAAGTCATCGTTATATCCAGCCCAACTTAATGG
**CsgF (N17D)**	Forward_end	GTGCTGCATATGCGTGTCAAACATGCAGTAGTTC
	Reverse_end	CCTTCAGGATCCTTAAAAATCGGTTGAGTTATTTTG
	Forward_intermediate	GGTGGTAACCCAGATAATGGCGCTTTTTTATTAAATAGC
	Reverse_intermediate	AAAAGCGCCATTATCTGGGTTACCACCAAAGTTTGGATTACG
**CsgF (N17Y)**	Forward_end	GTGCTGCATATGCGTGTCAAACATGCAGTAGTTC
	Reverse_end	CCTTCAGGATCCTTAAAAATCGGTTGAGTTATTTTG
	Forward_intermediate	GGTGGTAACCCATATAATGGCGCTTTTTTATTAAATAGC
	Reverse_intermediate	AAAAGCGCCATTATATGGGTTACCACCAAAGTTTGGATTACG

Constructs	Primers	Sequences
**CsgF (N17F)**	Forward_end	GTGCTGCATATGCGTGTCAAACATGCAGTAGTTC
	Reverse_end	CCTTCAGGATCCTTAAAAATCGGTTGAGTTATTTTG
	Forward_intermediate	AACTTTGGTGGTAACCCATTTAATGGCGCTTTTTTATTA
	Reverse_intermediate	TAATAAAAAAGCGCCATTAAATGGGTTACCACCAAAGTT
**CsgF (N17R)**	Forward_end	GTGCTGCATATGCGTGTCAAACATGCAGTAGTTC
	Reverse_end	CCTTCAGGATCCTTAAAAATCGGTTGAGTTATTTTG
	Forward_intermediate	GGTGGTAACCCACGTAATGGCGCTTTTTTATTAAATAGC
	Reverse_intermediate	AAAAGCGCCATTACGTGGGTTACCACCAAAGTTTGGATTACG
**CsgA**	Forward_end	GTGCTGCATATGAAACTTTTAAAAGTAGCAGCAATTG
	Reverse_end	CCTTCAGGATCCTTAGTACTGATGAGCGGTCGCG
**CsgA (△N22)**	Forward_end	GTGCTGCATATGAAACTTTTAAAAGTAGCAGCAATTG
	Reverse_end	CCTTCAGGATCCTTAGTACTGATGAGCGGTCGCG
	Forward_intermediate	CCGGTAGCGCTCTGGCATCTGAGCTGAACATTTAC
	Reverse_intermediate	GTAAATGTTCAGCTCAGATGCCAGAGCGCTACCGG
**CsgA (V2D)**	Forward_end	GTGCTGCATATGAAACTTTTAAAAGTAGCAGCAATTG
	Reverse_end	CCTTCAGGATCCTTAGTACTGATGAGCGGTCGCG
	Forward_intermediate	GGTAGCGCTCTGGCAGGTGATGTTCCTCAGTACGGCGGCGGC
	Reverse_intermediate	GCCGCCGCCGTACTGAGGAACATCACCTGCCAGAGCGCTACC

Constructs	Primers	Sequences
**CsgA (V3D)**	Forward_end	GTGCTGCATATGAAACTTTTAAAAGTAGCAGCAATTG
	Reverse_end	CCTTCAGGATCCTTAGTACTGATGAGCGGTCGCG
	Forward_intermediate	GGTAGCGCTCTGGCAGGTGTTGATCCTCAGTACGGC GGCGGC
	Reverse_intermediate	GCCGCCGCCGTACTGAGGATCAACACCTGCCAGAG CGCTACC
**CsgA (Q5A)**	Forward_end	GTGCTGCATATGAAACTTTTAAAAGTAGCAGCAATTG
	Reverse_end	CCTTCAGGATCCTTAGTACTGATGAGCGGTCGCG
	Forward_intermediate	CTGGCAGGTGTTGTTCCTGCGTACGGCGGCGGCGGT AACCAC
	Reverse_intermediate	GTGGTTACCGCCGCCGCCGTACGCAGGAACAACAC CTGCCAG
**CsgA (Y6A)**	Forward_end	GTGCTGCATATGAAACTTTTAAAAGTAGCAGCAATTG
	Reverse_end	CCTTCAGGATCCTTAGTACTGATGAGCGGTCGCG
	Forward_intermediate	CTGGCAGGTGTTGTTCCTCAGGCGGGCGGCGGCGG TAACCAC
	Reverse_intermediate	GTGGTTACCGCCGCCGCCCGCCTGAGGAACAACAC CTGCCAG
**CsgG**	Forward_end	GTGCTGCATATGCAGCGCTTATTTCTTTTGG
	Reverse_end	CCTTCAGGATCCTTAGGATTCCGGTGGAACCGACAT
**CsgG (I121D)**	Forward_end	GTGCTGCATATGCAGCGCTTATTTCTTTTGG
	Reverse_end	CCTTCAGGATCCTTAGGATTCCGGTGGAACCGACAT
	Forward_intermediate	TCTTTAACGGCGGCAAATGATATGGTTGAAGGTTCG ATT
	Reverse_intermediate	AATCGAACCTTCAACCATATCATTTGCCGCCGTTAAA GA

Constructs	Primers	Sequences
**CsgG (L172D)**	Forward_end	GTGCTGCATATGCAGCGCTTATTTCTTTTGG
	Reverse_end	CCTTCAGGATCCTTAGGATTCCGGTGGAACCGACAT
	Forward_intermediate	GTGAGTACCGGCGAGATCGATTCTTCGGTGAACACCAGT
	Reverse_intermediate	ACTGGTGTTCACCGAAGAATCGATCTCGCCGGTACTCAC
**CsgG (L236D)**	Forward_end	GTGCTGCATATGCAGCGCTTATTTCTTTTGG
	Reverse_end	CCTTCAGGATCCTTAGGATTCCGGTGGAACCGACAT
	Forward_intermediate	GATGGTATCGACCGTGGTGATTGGGATTTGCAAAATAAA
	Reverse_intermediate	TTTATTTTGCAAATCCCAATCACCACGGTCGATACCATC
**CsgG (W237D)**	Forward_end	GTGCTGCATATGCAGCGCTTATTTCTTTTGG
	Reverse_end	CCTTCAGGATCCTTAGGATTCCGGTGGAACCGACAT
	Forward_intermediate	GGTATCGACCGTGGTCTGGATGATTTGCAAAATAAAGCA
	Reverse_intermediate	TGCTTTATTTTGCAAATCATCCAGACCACGGTCGATACC
**CsgG (△W237)**	Forward_end	GTGCTGCATATGCAGCGCTTATTTCTTTTGG
	Reverse_end	CCTTCAGGATCCTTAGGATTCCGGTGGAACCGACAT
	Forward_intermediate	GGTATCGACCGTGGTCTGGATTTGCAAAATAAAGCA
	Reverse_intermediate	TGCTTTATTTTGCAAATCCAGACCACGGTCGATACC

Constructs	Primers	Sequences
**CsgG (△D238)**	Forward_end	GTGCTGCATATGCAGCGCTTATTTCTTTTGG
	Reverse_end	CCTTCAGGATCCTTAGGATTCCGGTGGAACCGACAT
	Forward_intermediate	ATCGACCGTGGTCTGTGGTTGCAAAATAAAGCAGAA
	Reverse_intermediate	TTCTGCTTTATTTTGCAACCACAGACCACGGTCGAT
**CsgG (△T31)**	Forward_end	GTGCTGCATATGCAGCGCTTATTTCTTTTGG
	Reverse_end	CCTTCAGGATCCTTAGGATTCCGGTGGAACCGACAT
	Forward_intermediate	ACCCATCTGCCAGCGCCGGGTAAAATCTTTGTTTCG
	Reverse_intermediate	CGAAACAAAGATTTTACCCGGCGCTGGCAGATGGGT
**CsgG (△G32)**	Forward_end	GTGCTGCATATGCAGCGCTTATTTCTTTTGG
	Reverse_end	CCTTCAGGATCCTTAGGATTCCGGTGGAACCGACAT
	Forward_intermediate	CATCTGCCAGCGCCGACGAAAATCTTTGTTTCGGTA
	Reverse_intermediate	TACCGAAACAAAGATTTTCGTCGGCGCTGGCAGATG
**CsgG (△107-110)**	Forward_end	GTGCTGCATATGCAGCGCTTATTTCTTTTGG
	Reverse_end	CCTTCAGGATCCTTAGGATTCCGGTGGAACCGACAT
	Forward_intermediate	GAAAACGGCACGGTTGCCATCCCGCTGCAATCTTTA
	Reverse_intermediate	TAAAGATTGCAGCGGGATGGCAACCGTGCCGTTTTC

Constructs	Primers	Sequences
**CsgF (R8A)**	Forward_end	GTGCTGCATATGCGTGTCAAACATGCAGTAGTTC
	Reverse_end	CCTTCAGGATCCTTAAAAATCGGTTGAGTTATTTTG
	Forward_intermediate	CATGACTTTCCAGTTCGCGAATCCAAACTTTGGTGG
	Reverse_intermediate	CATGACTTTCCAGTTCGCGAATCCAAACTTTGGTGG
**CsgF (N9A)**	Forward_end	GTGCTGCATATGCGTGTCAAACATGCAGTAGTTC
	Reverse_end	CCTTCAGGATCCTTAAAAATCGGTTGAGTTATTTTG
	Forward_intermediate	GACTTTCCAGTTCCGTGCGCCAAACTTTGGTGGTAAC
	Reverse_intermediate	GTTACCACCAAAGTTTGGCGCACGGAACTGGAAAGTC
**CsgF (N11A)**	Forward_end	GTGCTGCATATGCGTGTCAAACATGCAGTAGTTC
	Reverse_end	CCTTCAGGATCCTTAAAAATCGGTTGAGTTATTTTG
	Forward_intermediate	CAGTTCCGTAATCCAGCGTTTGGTGGTAACCCAAAT
	Reverse_intermediate	ATTTGGGTTACCACCAAACGCTGGATTACGGAACTG
**CsgF (F5D)**	Forward_end	GTGCTGCATATGCGTGTCAAACATGCAGTAGTTC
	Reverse_end	CCTTCAGGATCCTTAAAAATCGGTTGAGTTATTTTG
	Forward_intermediate	GGCTGGAACCATGACTGATCAGTTCCGTAATCCAAAC
	Reverse_intermediate	GTTTGGATTACGGAACTGATCAGTCATGGTTCCAGCC
**CsgF (F7D)**	Forward_end	GTGCTGCATATGCGTGTCAAACATGCAGTAGTTC
	Reverse_end	CCTTCAGGATCCTTAAAAATCGGTTGAGTTATTTTG
	Forward_intermediate	GAACCATGACTTTCCAGGATCGTAATCCAAACTTTG
	Reverse_intermediate	CAAAGTTTGGATTACGATCCTGGAAAGTCATGGTTC

Constructs	Primers	Sequences
**CsgF (F12D)**	Forward_end	GTGCTGCATATGCGTGTCAAACATGCAGTAGTTC
	Reverse_end	CCTTCAGGATCCTTAAAAATCGGTTGAGTTATTTTG
	Forward_intermediate	GTTCCGTAATCCAAACGATGGTGGTAACCCAAATAATG
	Reverse_intermediate	CATTATTTGGGTTACCACCATCGTTTGGATTACGGAAC
**CsgF (F21D)**	Forward_end	GTGCTGCATATGCGTGTCAAACATGCAGTAGTTC
	Reverse_end	CCTTCAGGATCCTTAAAAATCGGTTGAGTTATTTTG
	Forward_intermediate	CCAAATAATGGCGCTGATTTATTAAATAGCGCTCAG
	Reverse_intermediate	CTGAGCGCTATTTAATAAATCAGCGCCATTATTTGG
**CsgF (L22D)**	Forward_end	GTGCTGCATATGCGTGTCAAACATGCAGTAGTTC
	Reverse_end	CCTTCAGGATCCTTAAAAATCGGTTGAGTTATTTTG
	Forward_intermediate	CCAAATAATGGCGCTTTTGATTTAAATAGCGCTCAG
	Reverse_intermediate	CTGAGCGCTATTTAAATCAAAAGCGCCATTATTTGG
**CsgF (L23D)**	Forward_end	GTGCTGCATATGCGTGTCAAACATGCAGTAGTTC
	Reverse_end	CCTTCAGGATCCTTAAAAATCGGTTGAGTTATTTTG
	Forward_intermediate	CAAATAATGGCGCTTTTTTAGATAATAGCGCTCAGGCC
	Reverse_intermediate	GGCCTGAGCGCTATTATCTAAAAAAGCGCCATTATTTG

Supplementary Table 2. CryoEM data collection, refinement and validation statistics.

	**CsgFG complex (EMD-0841, 6L7A)**	**CsgFG-CsgA$_{N22}$ complex (EMD-0842, 6L7C)**
**Data Collection**		
**Electron microscope**	**Titan Krios**	**Titan Krios**
**Electron detector**	**K2-Submit camera**	**K2-Submit camera**
**Voltage (kV)**	**300**	**300**
**Pixel size**	**1.32**	**1.32**
**Defocus range (µm)**	**1.3-2.5**	**1.3-2.5**
**Electron dose (e^-/Å^2)**	**50**	**50**
**Images**	**1959**	**2430**
**3D Reconstruction**		
**Final particles**	**372680**	**139702**
**Resolution**	**3.38**	**3.34**
**B-factor (Å^2)**	**-168.8**	**-173.9**
**Model composition**		
**Peptide chains**	**18**	**27**
**R.m.s deviations**		
**Bonds (Å)**	**0.005**	**0.005**
**Angles (º)**	**0.777**	**0.797**
**Ramachandran plot**		
**Favored (%)**	**94.93**	**95.73**
**Outliers (%)**	**0**	**0**
**Validation**		
**Molprobity score**	**1.55**	**1.58**
**Clash score**	**4.54**	**3.85**
**Rotamer outliers (%)**	**0.42**	**1.20**

SUPPLEMENTARY FIGURES

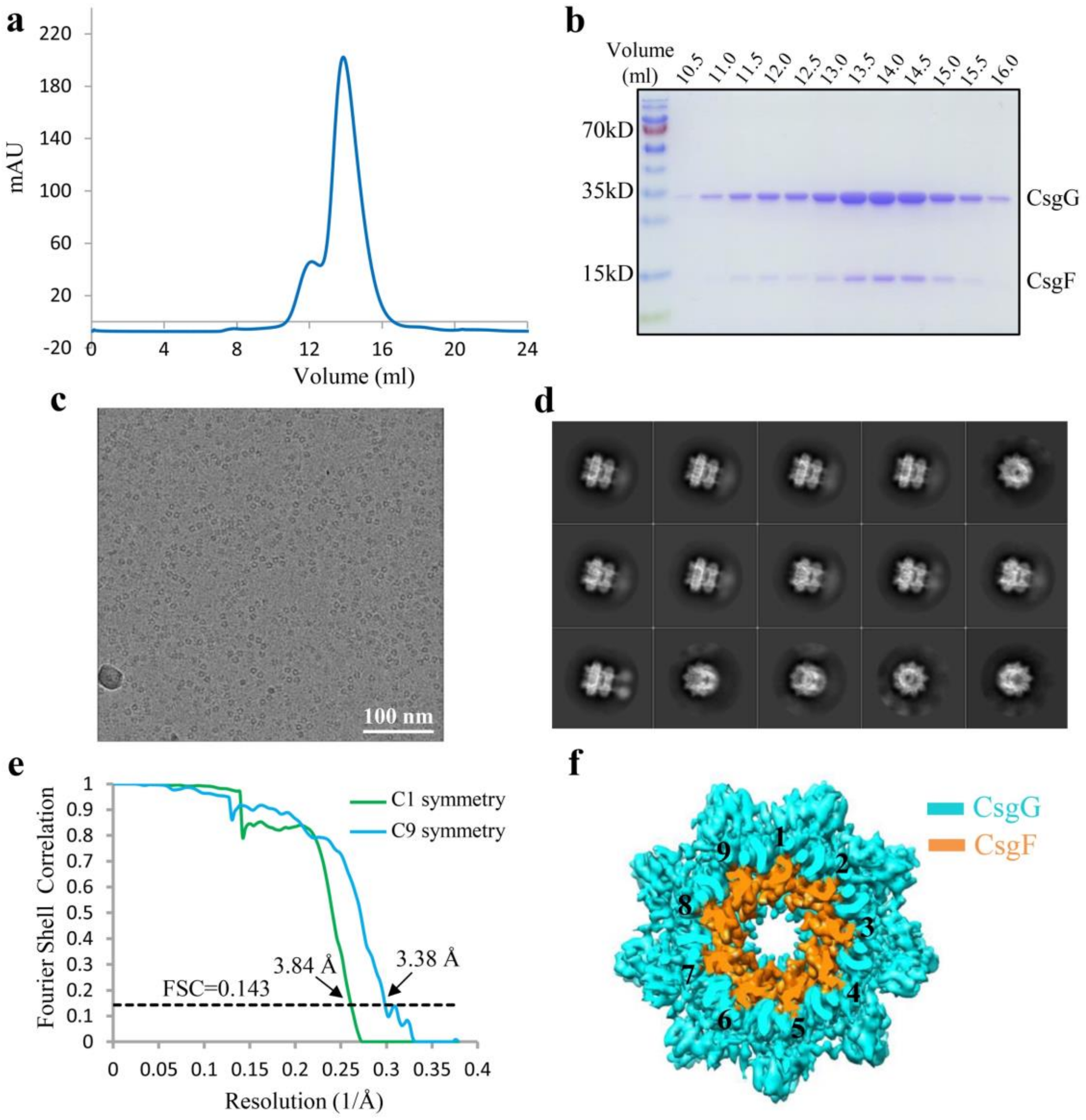

Supplementary Figure 1. Protein purification and cryoEM analysis of CsgFG. **a,** Size-exclusion chromatogram of the purified CsgEFG complex. **b,** SDS-PAGE analysis and Coomassie blue staining of the purified CsgEFG complex after size-exclusion chromatography. Source data are provided as a Source Data file. **c,** Representative cryoEM image of the CsgFG complex. Scale bar, 100 nm. **d,** Representative 2D classification of CsgFG complex. **e,** Gold standard Fourier shell correlation (FSC) curves of CsgFG complex reconstructed with C1 symmetry (green) and C9 symmetry (blue). **f,** Top-view of the CsgFG complex with C1 symmetry observed from the extracellular space. CsgG and CsgF are shown in cyan and orange, respectively.

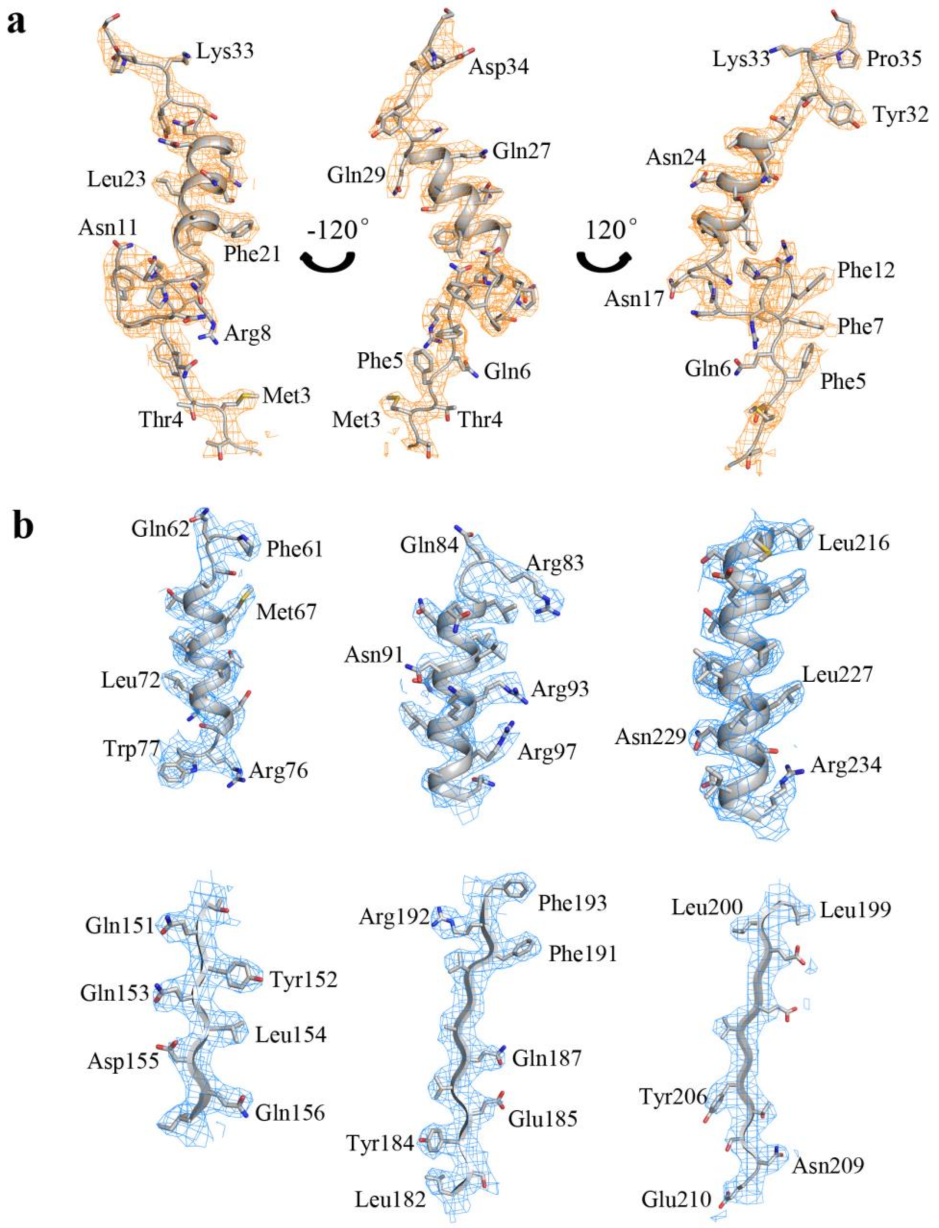

Supplementary Figure 2. The representative cryoEM densities at different regions of the CsgFG complex.

a, CryoEM density of $CsgF_N$ shown in different views. **b,** The representative cryoEM densities of CsgG residues with distinct side chains are labelled.

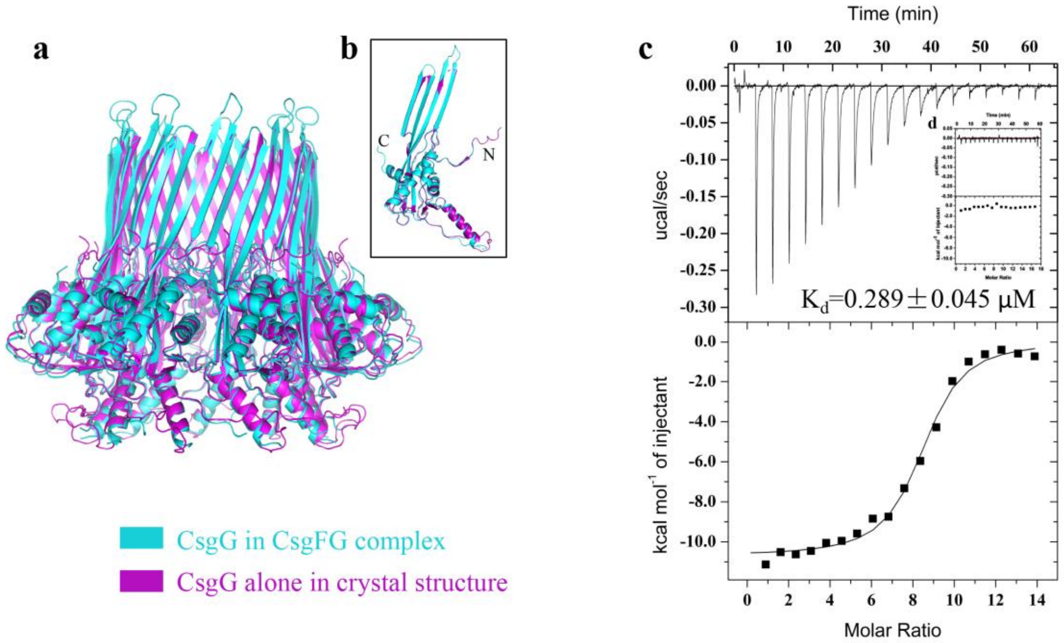

Supplementary Figure 3. Structural comparison of free CsgG and CsgF-bound CsgG and binding affinity between $CsgF_N$ and CsgG.

a, Structural comparison of nonamer CsgG in our CsgFG complex (cyan) and CsgG from others (purple, PDB code 4uv3)[1]. Both adopted similar folding patterns with 0.742 Å rmsd and showed no apparent difference. **b**, Structural comparison of monomer CsgG in our CsgFG complex (cyan) and CsgG from others(purple, PDB code 4uv3)[1]. **c**, The ITC profiles of the $CsgF_N$(C-terminal MBP tag) and CsgG nonamer. Source data are provided as a Source Data file. d, The ITC profiles of MBP and CsgG nonamer.

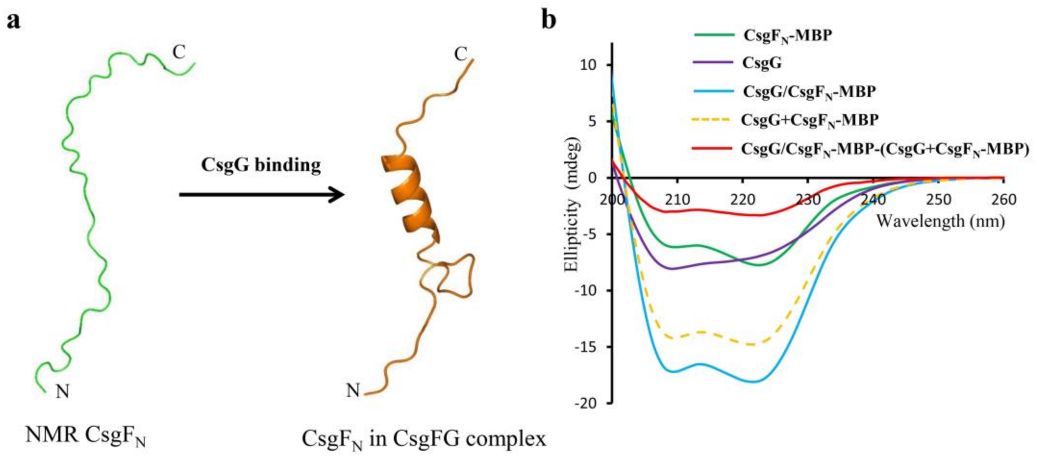

Supplementary Figure 4. Well folded structure of $CsgF_N$ in the CsgFG complex. **a**, Structural comparison of $CsgF_N$ between the reported NMR structure[2] and our structure in the CsgFG complex. The N- and C-termini are indicated. **b**, Circular Dichroism (CD) analysis for the $CsgF_N$ (C-terminal MBP tag) alone (green), CsgG alone (purple) and $CsgF_N$-CsgG complex together (blue). The sum of $CsgF_N$ alone and CsgG alone is shown by the orange dashed line. The difference between the experimental result of the $CsgF_N$-CsgG complex and the sum is shown in red, which was consistent with the typical CD spectra of the helix structure.

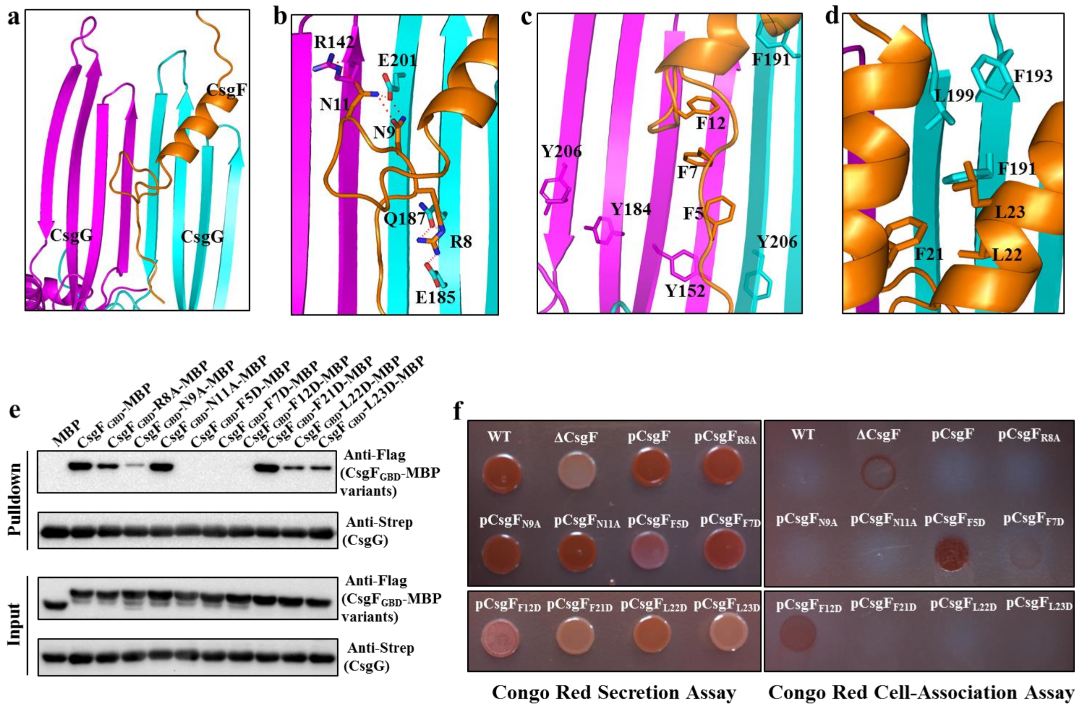

Supplementary Figure 5. Interaction mode between CsgF$_N$ and CsgG.

a, Interaction of CsgF$_N$ and CsgG. CsgF$_N$ is shown in orange. Two adjacent subunits of CsgG are shown in magenta and cyan, respectively. **b**, Hydrogen bonds and electrostatic interaction between CsgF$_N$ and CsgG. Hydrogen bonds are shown as red dashed lines. **c**, The folding of three aromatic residues in CsgF$_N$ and some typical aromatic residues in CsgG. **d**, The hydrophobic interaction between CsgF$_N$ and CsgG. **e**, Pulldown assay between the CsgF(C-terminal MBP tag) mutant variants and CsgG. Source data are provided as a Source Data file. **f**, Congo red secretion assay (left) and corresponding Congo red cell-association assay (right) for the CsgF mutant variants. Source data are provided as a Source Data file.

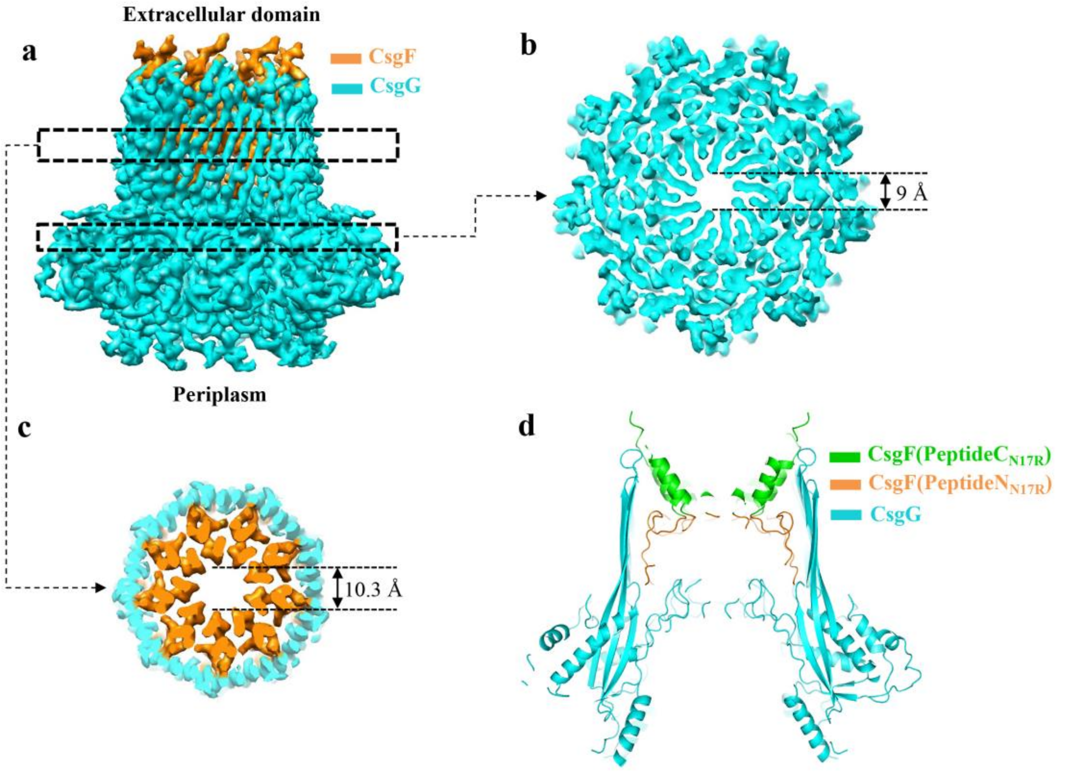

Supplementary Figure 6. Two-pore structure of the CsgFG complex.

a, Side view of the overall structure of the CsgFG complex. CsgG and CsgF are shown in cyan and orange, respectively. The positions of the two pores are indicated by black rectangles. **b**, Top view of the first pore structure formed by CsgG corresponding to the lower black rectangle in (**a**). **c**, Top view of the second pore structure formed by CsgF$_N$ corresponding to the upper black rectangle in (**a**)**. d**, Section view of the two-pore structure of CsgFG complex. PeptideN$_{N17R}$ is shown in orange and PeptideC$_{N17R}$ is shown in green.

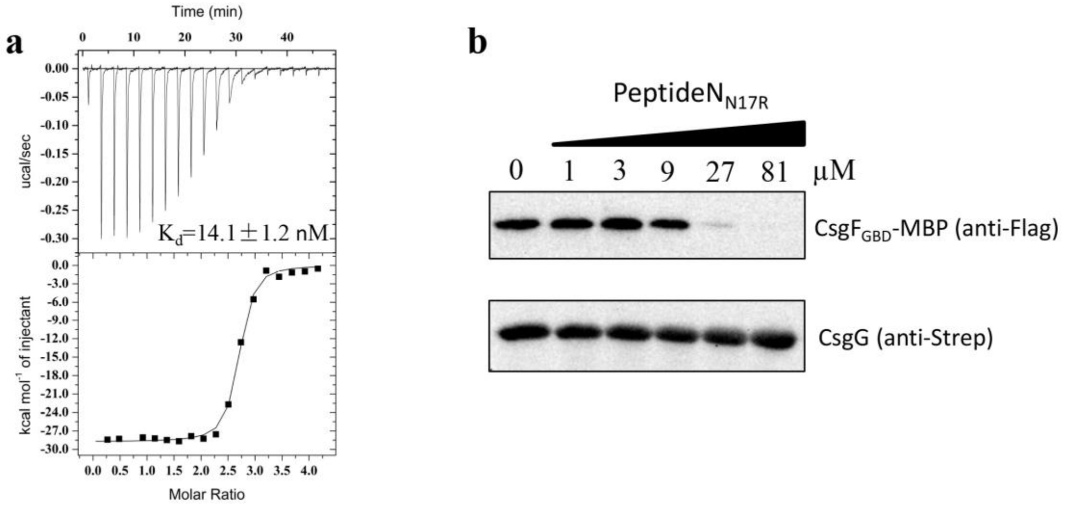

Supplementary Figure 7. Competitive binding of PeptideN$_{N17R}$ and CsgF.

a, The ITC profiles of the PeptideN$_{N17R}$ and CsgG nonamer. Source data are provided as a Source Data file. **b**, Competitive binding assay. CsgG is used as a bait to pulldown CsgF$_{GBD}$(C-terminal MBP tag) at different concentrations of PeptideN$_{N17R}$. Source data are provided as a Source Data file.

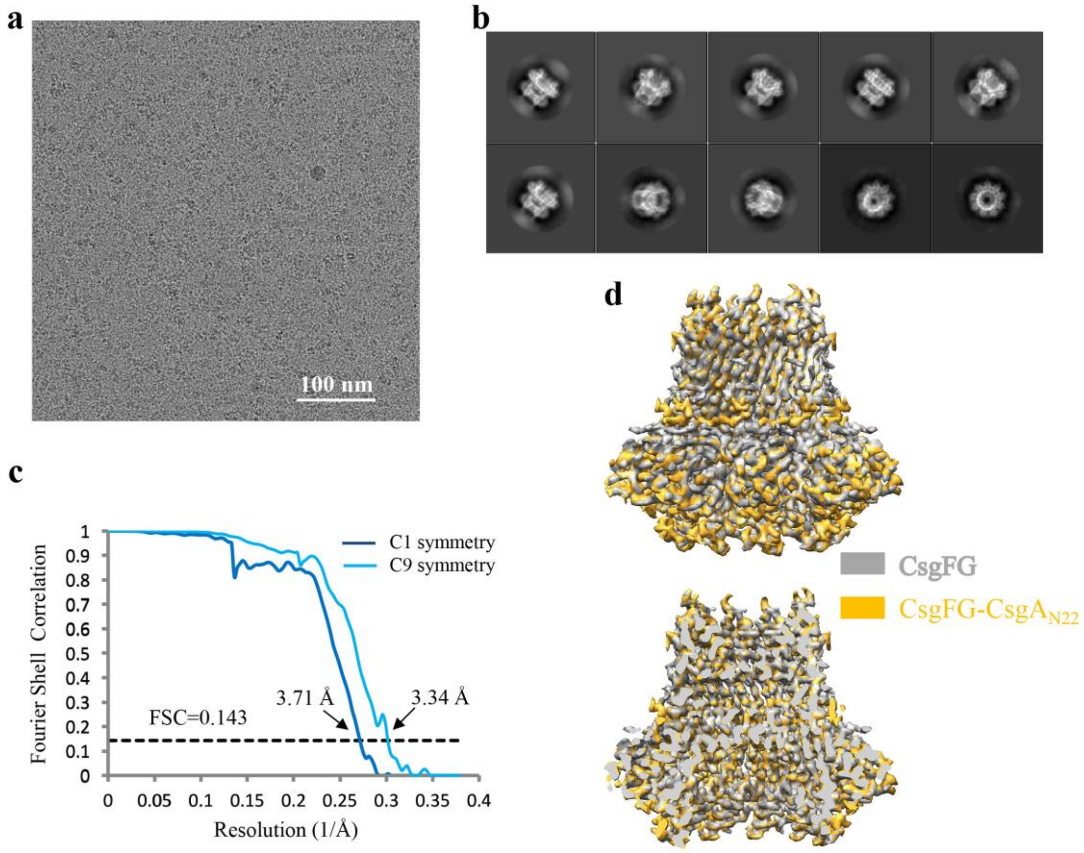

Supplementary Figure 8. CryoEM data analysis of CsgFG-CsgA$_{N22}$ complex.
a, Representative cryoEM image of the CsgFG-CsgA$_{N22}$ complex. Scale bar, 100 nm. **b**, Representative 2D classification of CsgFG-CsgA$_{N22}$ complex. **c**, Gold standard FSC curve of the CsgFG-CsgA$_{N22}$ complex. **d**, Structural comparison of the overall CsgFG (grey) and CsgFG-CsgA$_{N22}$ (yellow) complexes.

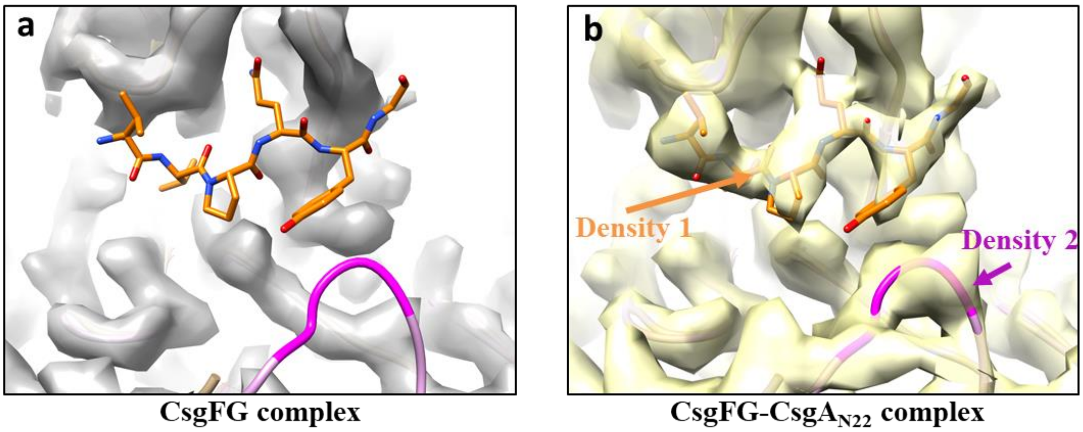

Supplementary Figure 9. Different cryoEM densities between the CsgFG and CsgFG-CsgA$_{N22}$ complexes.

a and **b** are density maps of the CsgFG complex and CsgFG-CsgA$_{N22}$ complex, respectively. The model of CsgFG-CsgA$_{N22}$ is overlapped with two maps. Two additional densities in **b**, corresponding to CsgA$_{N6}$ (orange model) and a CsgG loop region (purple model, residues 107 - 110), are shown and pointed by arrows, respectively.

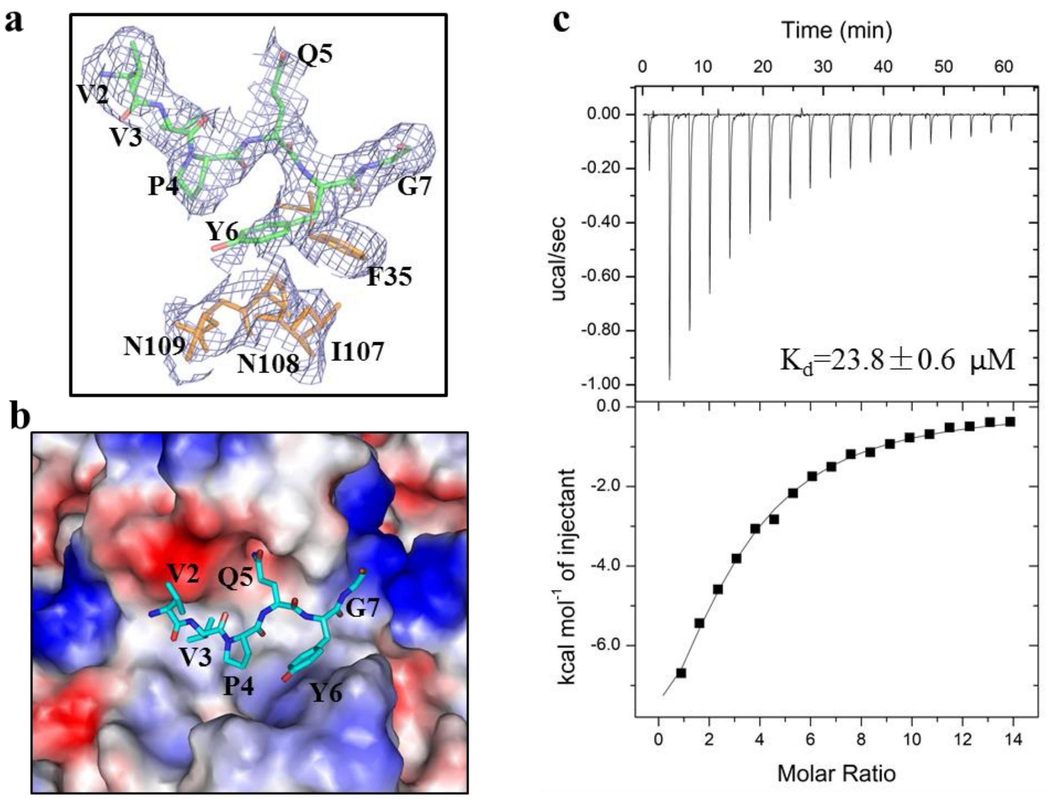

Supplementary Figure 10. Interaction between CsgA$_{N22}$ and the CsgG channel. **a**, CryoEM density of six residues of CsgA$_{N22}$. The corresponding model is shown in green chain and fit well into the additional density. A CsgG loop (I107 – N109) and several CsgG residues in background are drawn in orange. **b**, Six residues of CsgA$_{N22}$ in the crevice outside the CsgG channel. Electrostatic surface on the background is outside the CsgG periplasmic chamber. **c**, The ITC profiles of CsgA$_{N6}$ peptide and CsgG nonamer. Source data are provided as a Source Data file.

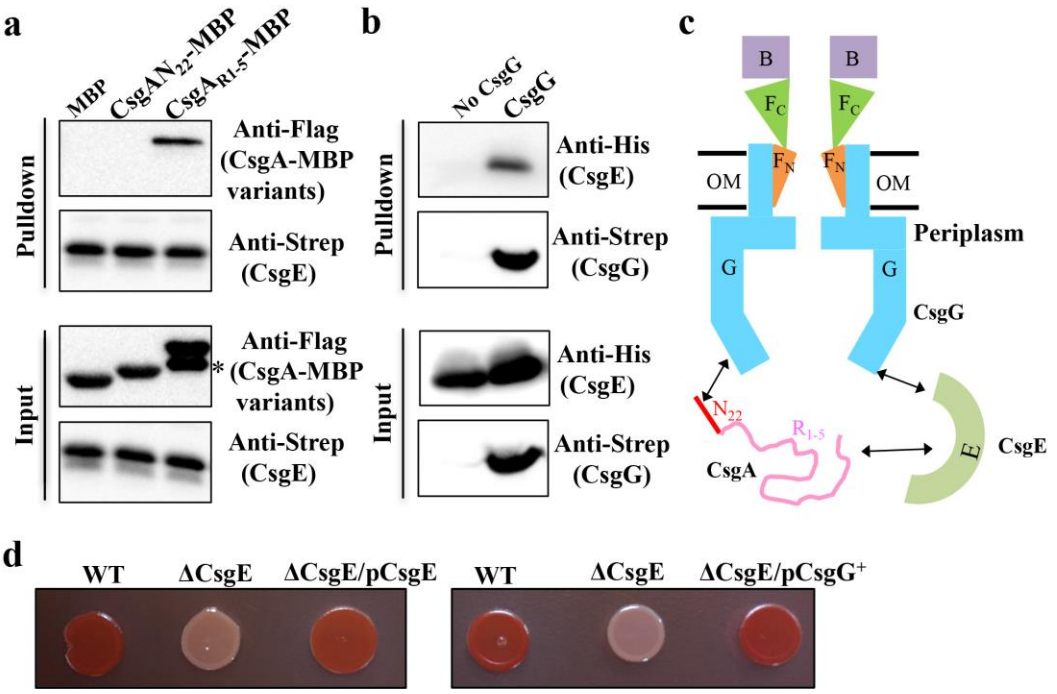

	Translocation efficiency per single CsgG	Number of CsgG channels	Amount of CsgA secretion	Congo red phenotype
Wild type	Normal	Normal	Normal	Red
Deletion of CsgE	Low	Normal	Reduced	White
Deletion of CsgE & over-expression of CsgG	Low	Increased	Normal	Red

Supplementary Figure 11. The interactions among CsgG, CsgE and CsgA and efficiency enhancing role of CsgE.

a, Pulldown assay of different parts of CsgA(C-terminal MBP tag) with CsgE. Source data are provided as a Source Data file. **b**, Pulldown assay of CsgE with CsgG. Source data are provided as a Source Data file. **c**, Schematic diagram for the triangular relationship between CsgA, CsgE, and CsgG. Interactions are indicated by black arrows. **d**, Congo red secretion assay of the CsgE deletion and CsgG over-expression in the *E.coli* BW25113 ΔCsgE strain. Source data are provided as a Source Data file. **e**, Explanation for Congo red phenotype in (**d**). CsgE deletion causes low translocation efficiency per CsgG channel while CsgG over-expression increases the number of CsgG channels.

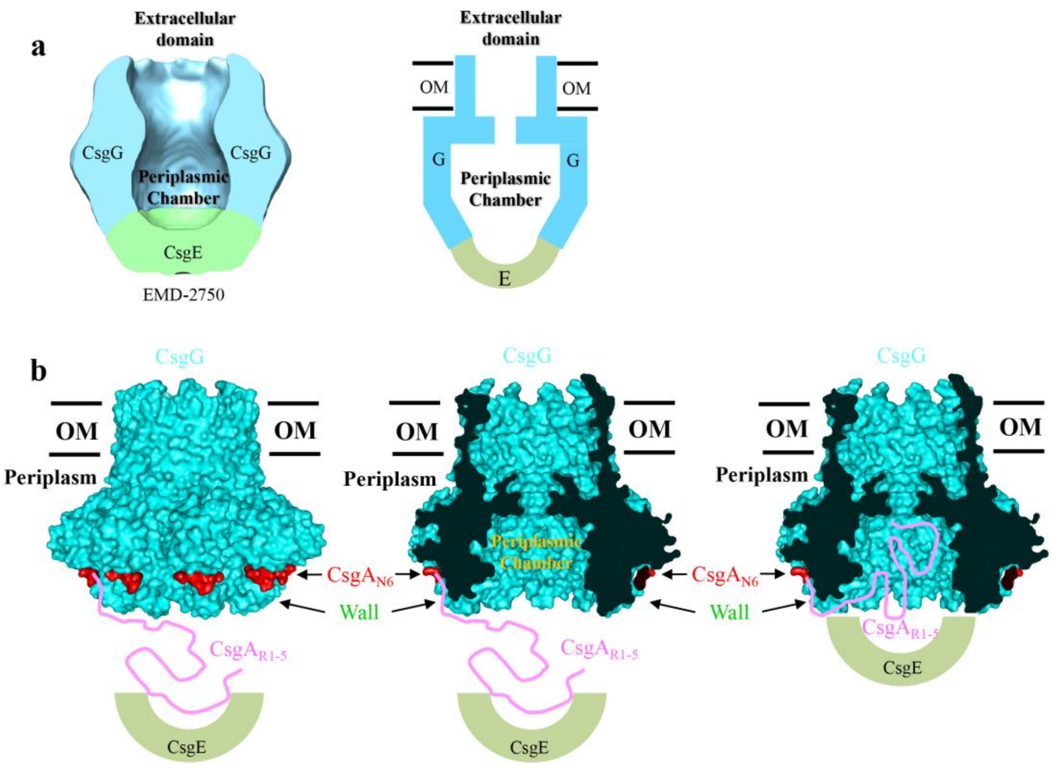

Supplementary Figure 12. Explanation of efficiency enhancing role of CsgE.

a, Side view of the previously reported cryoEM structure of CsgE-CsgG complex (left)[1] and the corresponding schematic diagram (right). Nine CsgEs form a cap-like structure (green map density) at the entrance to the periplasmic chamber of CsgG. **b**, Surface structure of CsgFG-CsgA$_{N22}$ complex and proposed role of CsgE. CsgA$_{N6}$ (red densities) is trapped on the crevice of CsgG surface and CsgE interacts with CsgA$_{R1-5}$ (left panel). Middle panel is the section view of left panel. CsgA$_{N6}$ is located at the back of periplasmic chamber and cannot access the periplasmic chamber. CsgE pushes CsgA$_{R1-5}$ into the periplasmic chamber of CsgG through the CsgE-CsgG interaction to enhance the translocation efficiency of CsgA (right panel).

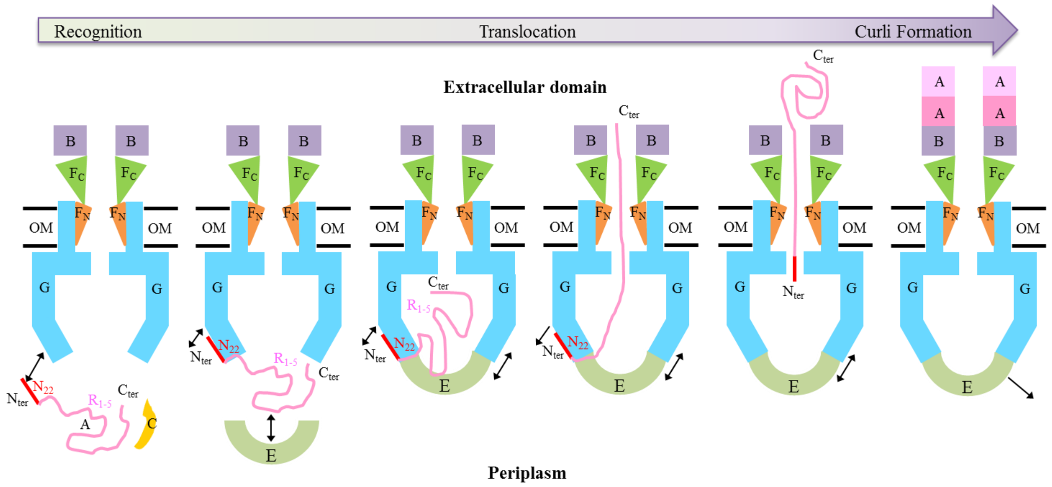

Supplementary Figure 13. Hypothesized CsgA recognition and secretion model of curli biogenesis system.

The six key steps that are hypothesized to occur during CsgA secretion across the CsgFG channel. First, the N22 sequence of CsgA binds to CsgG for CsgA recognition and enrichment near the CsgG channel. Second, CsgE binds to the $R_{1\text{-}5}$ repeats of CsgA. Third, CsgE carries $CsgA_{R1\text{-}5}$ to the large periplasmic chamber of CsgG through CsgE-CsgG interaction. Fourth, C-terminal CsgA is secreted by the pore structures of the CsgFG channel. Fifth, the N-terminus of CsgA dissociates from CsgG to allow further secretion of CsgA. Finally, CsgE dissociates from CsgG to enter into a new secretion cycle. During the progress in the periplasmic space, the chaperon protein CsgC stabilizes the soluble monomer CsgA. OM: outer membrane.

References

[1] Goyal, P. *et al.*, Structural and mechanistic insights into the bacterial amyloid secretion channel CsgG. *NATURE* **516** 250 (2014).

[2] Schubeis, T. *et al.*, Structural and functional characterization of the Curli adaptor protein CsgF. *FEBS LETT* **592** 1020 (2018).
